# Intakes and Food Sources of Dietary Fibre and Their Associations with Measures of Body Composition and Inflammation in UK Adults: Cross-Sectional Analysis of the Airwave Health Monitoring Study

**DOI:** 10.3390/nu11081839

**Published:** 2019-08-08

**Authors:** Rachel Gibson, Rebeca Eriksen, Edward Chambers, He Gao, Maria Aresu, Andrew Heard, Queenie Chan, Paul Elliott, Gary Frost

**Affiliations:** 1 Section for Nutrition Research, Faculty of Medicine, Imperial College, London W12 0NN, UK; 2 Department of Nutritional Sciences, School of Life Course Sciences, King’s College London, London SE1 9NH, UK; 3 MRC-PHE Centre for Environment and Health, Department of Epidemiology and Biostatistics, School of Public Health, Imperial College, London W2 1PG, UK; 4 NIHR Imperial College London Biomedical Research Centre, Exhibition Road, London SW7 2AZ, UK; 5 Dementia Research Institute, Imperial College London, Hammersmith Hospital campus, Du Cane Road, London W12 0NN, UK; 6 Health Data Research UK London, Imperial College London, Exhibition Road, London SW7 2AZ, UK

**Keywords:** dietary fibre, food sources fibre, body mass index, body composition, waist circumference, C-reactive protein, Airwave Health Monitoring Study, UK population

## Abstract

The purpose of this study was to investigate the associations between intakes of fibre from the main food sources of fibre in the UK diet with body mass index (BMI), percentage body fat (%BF), waist circumference (WC) and C-reactive protein (CRP). Participants enrolled in the Airwave Health Monitoring Study (2007–2012) with 7-day food records (*n* = 6898; 61% men) were included for cross-sectional analyses. General linear models evaluated associations across fifths of fibre intakes (total, vegetable, fruit, potato, whole grain and non-whole grain cereal) with BMI, %BF, WC and CRP. Fully adjusted analyses showed inverse linear trends across fifths of total fibre and fibre from fruit with all outcome measures (*p_trend_* < 0.0001). Vegetable fibre intake showed an inverse association with WC (*p_trend_* 0.0156) and CRP (*p_trend_* 0.0005). Fibre from whole grain sources showed an inverse association with BMI (*p_trend_* 0.0002), %BF (*p_trend_* 0.0007) and WC (*p_trend_* 0.0004). Non-whole grain cereal fibre showed an inverse association with BMI (*P_trend_* 0.0095). Direct associations observed between potato fibre intake and measures of body composition and inflammation were attenuated in fully adjusted analyses controlling for fried potato intake. Higher fibre intake has a beneficial association on body composition, however, there are differential associations based on the food source.

## 1. Introduction

The prevalence of adult overweight and obesity is continuing to rise; it is estimated that ~58% of the global population will be overweight or obese by 2030 [1]. In addition to the impact of obesity at an individual level in terms of morbidity, the global economic burden of obesity is estimated to be 2.8% of gross domestic product [2]. Evidence supports that positive energy balance has a direct association with body mass [3] with diet and physical activity established as modifiable factors in the trajectory of adult weight gain [4]. In turn, excess body fat, specifically excess visceral fat, is an essential component of the pathophysiology of cardiometabolic disease [5]. Understating the modifiable factors to prevent excess adiposity is, therefore, a public health priority.

Dietary fibre is a heterogeneous group of compounds consumed from a variety of plant food sources. Existing research has focused on total fibre intakes and suggests an inverse association between total dietary fibre and body weight [6]. Limited studies have considered the food sources of fibre. Where there is evidence, studies have observed differential benefits of fibre intake from cereal, fruit and vegetable sources on obesity-related cardiometabolic health outcomes [7,8,9,10]. Understanding the relationship between food sources of fibre and body composition may be more readably translated to food-based eating guidelines. A limitation of previous studies exploring fibre intakes is the use of food frequency questionnaire (FFQ) data collection methods [11], rather than prospective 7-day estimated weighed diet records. Compared to FFQs, 7-day estimated weighed dietary records have been shown to have a greater agreement with dietary fibre intake collected from 16-day weighed records (gold standard method) [12]. The aim of this study was to investigate the associations of dietary fibre intakes from major UK food sources of fibre: potatoes, cereal (whole grains and non-whole grain cereal), fruit, vegetables, and legumes with measures of body composition (body mass index, waist circumference, and total body fat) and C-reactive protein—a marker associated with abdominal adiposity [13] and a strong predictor of future cardiovascular disease (CVD)risk [14,15]. This study was conducted in a large UK occupational cohort, the Airwave Health Monitoring Study—an ongoing longitudinal study of British police force employees [16].

## 2. Materials and Methods

### 2.1. Study Population

Recruitment procedures and baseline characteristics of the Airwave Health Monitoring Study of the British police forces have been described previously [17]. Dietary data from a random sample of food diaries collected between 2007 and 2012 (*n* = 7771) were used for the present study. We excluded participants with self-reported chronic disease diagnosis at enrolment: angina, heart disease, chronic obstructive pulmonary disease, cancer, chronic liver disease, thyroid disease, arthritis, diabetes (type 1 or type 2) and/or previous stroke (*n* = 501) as these diseases may affect dietary intakes. Participants were excluded based on missing data for primary outcomes. No female participant reported being pregnant. The final sample size included in the present study was 6898 (Supplemental Figure S1). The Airwave Health Monitoring Study is conducted according to the guidelines laid down in the Declaration of Helsinki and all procedures involving human subjects were approved by the National Health Service Multi-Site Research Ethics Committee (MREC/13/NW/0588). Written informed consent was obtained from all participants.

### 2.2. Assessment of Fibre Intake and Other Dietary Variables

Dietary intake was measured using 7-day estimated weighed food diaries. Calculation of nutritional intake was conducted using Dietplan software (Forestfield Software Ltd, Horsham, UK) which was based on the McCance and Widdowson’s 6th and 7th Edition Composition of Foods UK Nutritional Dataset (UKN) following a study-specific standard protocol [17]. To account for individual differences in reporting and total energy intake, energy-adjusted dietary variables were calculated using the nutrient density method [18]. The Goldberg method was applied to estimate prevalence of energy intake misreporting [19], the methods and results of which have previously been reported in detail [17]. Fibre intake was estimated from the American Association of Analytical Chemists (AOAC) analytical method of dietary fibre reported in the UKN database. Fibre intake from the following food groups of interest were calculated: *i)* fruits, *ii)* vegetables (excluding legumes and white potatoes), *iii)* potatoes (excluding sweet potato), *iv)* legumes (including peanuts), *v)* whole grains, *vi)* nuts and seeds and *vii)* non-whole grain cereal sources. Fruit and vegetable classifications were based on common UK culinary usage, e.g., tomato as a vegetable, sweet corn as vegetable. Whole grain content of foods was estimated from previously published data [20] and on-line manufacturer declarations. Table S1 details the foods classified in each food group.

### 2.3. Measures of Body Composition

Enrolled participants attended a regional health-screening clinic. Trained research nurses used a standard protocol to conduct all clinical examinations as described previously [16]. The primary outcome measurements for the current analyses were body mass index (BMI), percentage body fat (%BF), waist circumference (WC) and C-reactive protein (CRP). Body weight was measured to the nearest 0.05 kg using digital scales (Marsden digital weighing scale). Standing height was measured to the nearest 0.1 cm (Marsden H226 portable stadiometer, Marsden Weighing Group, South Yorkshire, UK). BMI was calculated as weight (kg)/height(m^2^), %BF was measured via bioelectrical impedance analysis (Tanita BC-418MA body composition analyser, Tanita Corp., Tokyo, Japan), WC was measured between the lower rib margin and the iliac crest in the mid-axillary line using a Wessex-finger/joint measure tape (Seca 201, Seca Ltd, Birmingham, UK) and CRP was measured using serum (IL 650 analyser Instrumentation Laboratory, Bedford, MA, USA).

### 2.4. Measurement of Covariates 

Data on occupational, lifestyle, medical history, socioeconomic and demographic factors were collected during the health-screen visit using a structured on-line questionnaire. Total working hours (including usual weekly overtime) was classified into categories (<40, 41–48, ≥49 hours per week) [21,22]. Physical activity information was collected using The International Physical Activity Questionnaire Short Form (IPAQ-SF) [23] which calculates metabolic equivalent minutes per week across three exercise parameters (walking, moderate and vigorous) with participants categorised as undertaking a high, moderate or low level of activity [24]. Weekly TV viewing time was recorded as part of the lifestyle questionnaire in multiples of 15 minutes and categorised into three groups (high, moderate and low) based on tertile cut-off values (TV viewing hours per week: low <6, moderate 6–15, high >15).

### 2.5. Statistical Analyses 

To assess differences between two groups, independent *t*-tests were used for data with a normal distribution (mean and standard deviation presented) and Mann-Whitney U-tests were used otherwise (median and interquartile range presented). To achieve a normal distribution CRP was logarithmically transformed. Associations across categorical variables were analysed using Chi-Squared test (χ²). Fibre from legumes was combined with fibre from vegetables for the analyses due to low legume intake. Food sources of fibre intake data were highly skewed. Therefore, general linear models tested the association between fifths of fibre intake (g/ 1000 kcal) and each outcome variable of interest. Table S2 presents the cut-off intakes by quantile. To test linear associations, orthogonal polynomial coefficients were generated and applied to correct for the unequal spacing between median values of each quintile of intake [25]. Adjusted means are presented with corresponding 95% confidence interval (95% CI). Two models were constructed for the analyses and adjusted for confounders. Covariates were selected for inclusion into the models by either *i)* an observed significant statistical association (*p* < 0.05) with both the independent variable and dependent variable under investigation (and plausibly classified as a confounder) or *ii) a priori* based on an association determined in previous cohort studies. The crude model was adjusted for age (continuous) and sex. The fully adjusted model was adjusted additionally for ethnicity, marital status, final attained educational level, length of weekly working hours, smoking (current, previous, never), daily TV viewing hours (thirds) and physical activity (IPAQ category), alcohol intake (mean g/day), total energy, and macronutrients: saturated fat, polyunsaturated fat, non-milk extrinsic sugars (all % energy intake), and sources of fibre other than those under study (g/1000 kcal). Based on previous studies indicating an association between fried potato consumption with cardiometabolic risk [26,27] we additionally adjusted the models for potato fibre by categories of fried potato consumption (nil consumers, low consumers, and high consumers who recorded below/above sex-specific median intakes when non-consumers removed: men 22.4 g/day, women 17.8 g/day). Statistical analyses were conducted using SAS version 9.4 (SAS Institute, Cary, NC, USA). Statistical tests were two-sided with a significance level at 0.05.

### 2.6. Additional Analyses

Three sets of stratified analyses were conducted. Firstly, as obesity may lie on the causal pathway between diet and measures of inflammation, we stratified by BMI (<25 kg/m^2^ and ≥25 kg/m^2^). Secondly, as carbohydrate intake is associated with dietary fibre intakes and may potentially modify associations between fibre and measures of body composition, we stratified by low and high carbohydrate intake. High carbohydrate intake defined as energy intake of ≥50% derived from carbohydrate; this value identifies those above the mean intake within the cohort and is also the guideline amount for the UK population. Lastly, we stratified participants by those estimated to be acceptable and under-reporters of energy intake to test the robustness of our results against potential dietary intake misreporting [28]. We also conducted linear regression analyses with transformed independent variables (fibre intakes) to estimate beta coefficients.

## 3. Results

### 3.1. Cohort Characteristics

Male employees accounted for 61.2% of the sample; mean age was 41.1 standard deviation (SD) 9.1 years, and 75.2% were employed in England. The majority of the cohort had a BMI above 25 kg/m^2^ (66.6%), and 49% had a waist circumference higher than sex-specific cut-off values (Table 1). Mean daily fibre intake for the cohort was 17.3 SD 6.0 g and 3.3% of participants had mean intakes of 30 g or more per day. The main sources of fibre intake were non-whole grain cereal sources (39.9 SD 12.7%), vegetables excluding legumes (16.2 SD 8.6%), and potato (13.6 SD 8.6%) (Table 2). Sources of fibre differed across total fibre intake categories; with participants in the lowest category obtaining 47.8% (SD 12.7%) of fibre from non-whole grain cereal sources and 13.8% (SD 9.2%) from potatoes compared to 30.9% (SD 11.2%) and 8.2% (SD 6.0%), respectively, for participants in the highest fifth of total fibre intake (Table S3). Participants in the lowest fifth of total fibre intake were more likely to be male, employed in Scotland and work more than 40 hours per week; they were also less likely to have obtained a degree or postgraduate qualifications (Table S3).

### 3.2. Fibre Intake and Body Composition 

After adjustment for potential confounders, including energy intake, there were significant inverse linear trends for all measures of body composition across fifths of total and fruit fibre intakes (Table 3). A direct association was observed between fibre from potatoes and all measures of body composition in the crude models (adjusted for age and sex). Full adjustment for confounders attenuated these associations. Fibre from non-whole grain cereal sources showed an inverse association with BMI but no association with %BF, WC or CRP in fully adjusted models. Fibre from vegetables and legumes showed no association with %BF or BMI; however, inverse associations were observed with WC (*p*_trend_ = 0.0156) and CRP (*p*_trend_ = 0.005). Tests of linear association conducted using linear regression (Table S4) showed comparable associations, with total and fruit fibre inversely associated with all outcome measures.

### 3.3. Additional Analyses

For total fibre intakes, trend estimates were comparable across analyses stratified by BMI (</≥25 kg/m^2^). In predicted under-reporters of energy intake, the significance between fibre intake and CRP was attenuated in the fully adjusted model. In participants with high carbohydrate intake (≥50% EI from carbohydrate), there was an attenuation of the relationship between total fibre intake and CRP and BMI (Tables S5–S10).

## 4. Discussion

### 4.1. Summary

Few studies have investigated the associations between fibre intakes from different food sources with measures of body composition. In this large UK population sample, we demonstrate that total fibre and fruit fibre intakes are inversely associated with all measures of body composition while potato fibre shows no association. Our findings suggest an inverse dose–response trend between total fibre intake with body composition and inflammation. The latter is supported by a previous longitudinal study in a US population sample that observed a 63% lower risk of elevated C-reactive protein (CRP) in the highest versus lowest quartile of total fibre intake [29]. Although our findings support a previous meta-analysis of an inverse association between total dietary fibre intake and cardiometabolic risk [30], there are limited large observational studies to compare our findings with. A previous study in a Spanish adult population sample found that lower total fibre intake was associated with overweight and obesity [31]. However, in contrast to our findings, the association did not remain significant when only plausible energy reporters were analysed [31].

In common with other nutrients, dietary fibre is obtained from several different food sources (fruit, vegetables and grains), all of which contain differing nutrient profiles. By testing associations between the main UK food sources of dietary fibre, we have been able to estimate independent associations of food sources of fibre on measures of body composition. Fruit fibre was the only source of fibre that was consistently inversely associated with all four measures of body composition, while whole grain sources of fibre were inversely associated with three, and vegetables (including legumes) with two. Non-whole grain sources of fibre only showed an inverse association with BMI. Although differential effects of food sources of fibre on health outcomes have been previously observed, there are a limited number of studies exploring associations with body composition. Pooled prospective data from European Prospective Investigation into Cancer and Nutrition (EPIC)-InterAct study indicated fibre from cereals but not from fruit and vegetables combined was inversely associated with overall and abdominal fat gain [10], while a further study in a Dutch population sample observed cereal fibre to be only inversely associated with BMI in men [9]. In terms of cardiometabolic health, fibre from cereals and vegetables but not from fruit were associated with reduced incidence of type 2 diabetes risk [8], while a meta-analysis of prospective studies reported fruit fibre to be associated with reduced risk of cardiovascular disease [30]. The Nurses’ Health Study and Health Professionals Follow-up Study reported no association across quintiles of total fibre intake and risk of coronary heart disease, while observing a protective dose–response effect from cereal fibre [11]. The apparent difference from our findings may relate to difference in outcomes, with our study considering body composition, an intermediate risk factor, compared to cardiometabolic disease end points. We also used prospective compared to retrospective dietary assessment and we separated whole grain and non-whole grain cereal fibre.

In minimally adjusted models we show a direct association between potato fibre intake with body composition, however, this significance was attenuated when analyses were adjusted for additional confounders including saturated fat intake and cooking method (frying). Given that potato consumption is an important component of total fibre intake (contributing to ~14% of total fibre intake in the Airwave Health Monitoring Study population sample) and recent research has reported that potato consumption to be associated with an increased risk of hypertension [32] and type 2 diabetes [26], the lack of an association between potato fibre and body composition needs further investigation. In fully adjusted analyses we observed fibre from non-whole grain cereal sources (the main contributor to fibre intake) to only remain inversely associated with BMI after adjustment for confounders. Non-whole grain cereal and potato food sources may be subject to more extensive processing than other food groups in terms of preparation (e.g., potatoes require cooking before consuming and non-whole grain cereal grains are commonly used in baked goods such as biscuits and cakes). Levels of processing can influence the fibre composition of starchy foods, for example, retrograded amylase and starch (resistance starch III [RSIII]) is a digestion resistant carbohydrate formed through heating and cooling of starchy foods [33]. No estimates exist for the intake of RSIII from different food sources in the UK population and further evidence is needed to determine if RS exert different changes on the pathways associated with body composition compared to native fibres. 

### 4.2. Potential Mechanisms 

The mechanisms linking dietary fibre and body composition are yet to be fully characterised. Controlled human feeding studies supplementing with fibre have observed beneficial outcomes on weight loss through increased satiety and a decrease in hunger therefore, lowering overall energy intake [34,35]. Both of these studies supplemented with oligofructose, a fibre that is predominately obtained from wheat products in the Western diet [36], supporting our observed association of higher whole grain and non-whole grain cereal fibre intakes with lower BMI. However, fibre from whole grain sources, but not non-whole grain sources, was associated with lower waist circumference and percentage of body fat, suggesting that some benefits of fibre from whole grains are attributable to nutrients and non-nutrient components (e.g., phytochemicals) linked to whole grain fibre. A potential mechanism for the differential effects of food sources of fibre on markers of body composition and inflammation is via gut microbiota profile modification in response to different types of fibres (e.g., cellulose, lignin) combined with bioactive compounds such as polyphenols [37] contained in different food sources. Furthermore, differential products from dietary fibre fermentation by bacteria in the colon, such as short-chain fatty acids, have been shown to impact on metabolic pathways [38]. In interpreting our observations regarding the food source of fibre, it is important to acknowledge that the food source of fibre may simply be a marker for other dietary components. Moreover, other nutrients consumed with the fruit, or indeed the lower energy density of fruit compared to some other foods may mean that eating fruit simply displaces other more caloric foods.

### 4.3. Public Health Nutrition Implications

It is estimated that about nine percent of the UK adult population meet the target intake for dietary fibre [39], which is more than we observed in British police force employees with <5% consuming the 30 g or more a day recommendation. We have previously reported that specific occupational factors in the Airwave Health Monitoring Study Cohort to be associated with a poorer diet quality—duration of weekly working hours and job strain [40], which may help explain their lower fibre intakes. The low intake of whole grains and lack of specific whole grain guidelines in the UK has previously been emphasised [20,41]. The observation that higher whole grain consumption was associated with increased overall fibre intake and that it was independently associated with important markers of cardiometabolic risk, supports the need for clearer population guidelines to improve whole grain intakes such as the Dutch food-based dietary guidelines [42]. Our finding that fruit sources of fibre intake are consistently inversely associated with measures of adiposity and inflammation is aligned with prospective evidence that demonstrates lower overweight in persons that consume more fruit [43].

### 4.4. Strengths and Limitations

The main strength of our study is that we estimated fibre intakes from 7-day food records, shown to provide improved estimations of fibre intake compared to food frequency questionnaires [12]. Additionally, our application of extensive food group disaggregation to the dietary dataset has facilitated the investigation of fibre intakes from a wide range of food sources. Another strength is the objective measure of body composition as part of a rigorous clinical protocol [16]. Although the sample included in the study was drawn from a specific occupational group, potentially limiting generalizability of findings, the period of greatest weight gain in adulthood occurs between the ages of 25–45 years [44]; therefore, it is of public health interest to understand how specific dietary exposures associate with body composition at this key life stage. Additionally, this study included a high proportion of males in early adulthood who are under-represented in existing UK cohort studies [45]. The established limitation of all current dietary measurement tools needs to be acknowledged—the reliance on self-report. We have previously reported the estimated prevalence of energy intake underreporting to be comparable to national UK diet survey data and biased towards participants with higher BMI [17]. We, therefore, conducted energy-adjusted analyses and, to investigate the effect of differential error, we stratified participants by energy reporting status. Absolute intakes of fibre may be underreported along with energy intake. However, the mean fibre intake for those classed as acceptable energy intake reporters was 19 g/day (vs. the cohort average of ~17 g/day)—a value that is still considerably lower than UK recommendations of 30 g/day. The overall trends in energy-adjusted intakes against objective measures of body composition are less likely to be influenced by errors in absolute energy intake reporting. It is not possible to estimate reporting errors at the food level. Underreporting may not be distributed equally across all types of foods but may be biased towards ‘unhealthy’ foods [46] and, therefore, overestimation of fibre per unit of energy intake is a possibility. As with all observational nutritional epidemiological studies, understanding the effect of an individual component in the diet against overall dietary intake is challenging due to collinearity between dietary components. Additionally, the observed associations reported in this study may simply be due to uncontrolled residual confounding, either in variables not controlled for or, because of imprecision in using self-reported and categorised data to estimate covariates. Lastly, the cross-sectional nature of our study limits any causal inferences that can be derived from our data.

## 5. Conclusions

In conclusion, this study contributes to the limited existing evidence base on the beneficial associations between dietary fibre and body composition. In the face of the current trend to focus on the macronutrient content of our diet, the importance of dietary fibre to health is too often neglected. It is important that public health nutrition practitioners continue to work to increase fibre intakes in the UK population; one potential avenue would be the promotion of low consumed food groups such as legumes and whole grains in addition to fruit and vegetables in keeping with broader nutritional recommendations.

## Figures and Tables

**Table 1:** Characteristics of the Airwave Health Monitoring Study participants with dietary data available 2007–2012 (*n* = 6898).

Characteristic	Mean	SD
**Age, years (SD)**	41.1	9.1
	**N**	**%**
**Men N (%)**	4220	61.2
**White**	6707	97.3
**Relationship status**		
**Cohabiting**	1125	16.7
**Divorced/separated**	540	8.0
**Married**	4308	64.0
**Single**	763	11.3
**Missing**	162	2.3
**Education**		
**Left school before taking GCSE**	259	3.8
**GCSE or equivalent**	2049	29.7
**Vocational qualifications**	498	7.2
**A-levels/Highers or equivalent**	2218	32.2
**Bachelor degree or equivalent**	1442	20.9
**Postgraduate qualifications**	431	6.3
**Employment force, country**		
**England**	5175	75.2
**Scotland**	1093	15.9
**Wales**	614	8.9
**Missing**	16	<0.01
**Rank**		
**Police Constable/Sergeant**	433	7.7
**Inspector and above**	3295	58.6
**Police staff/Other**	1895	33.7
**Missing**	1275	18.4
**Hours worked per week**		
**<35**	596	8.6
**35 < 40**	2637	38.2
**40 < 49**	2260	32.8
**49 < 55**	726	10.5
**55+**	679	9.8
**Physical activity ^a^**		
**Low**	772	11.2
**Moderate**	3087	44.8
**High**	3039	44.1
**Smoking status**		
**Never smoker**	4780	69.5
**Former smoker**	1542	22.4
**Current smoker**	554	8.1
**Missing**	22	
**Body Mass Index**		
**<25 kg/m^2^**	2303	33.4
**≥25 kg/m^2^ and <30 kg/m^2^**	3288	47.7
**>30 kg/m^2^**	1307	18.9
Waist circumference risk category *		
**Healthy measurement**	3513	51.0
**Elevated risk**	3385	49.0
**C-reactive protein**		
**<1 mg/L**	3715	53.9
**≥1 mg/L**	3183	46.1

GCSE general certificate secondary education. SD Standard deviation a. Physical activity determined by classification of metabolic equivalents. *Sex-specific cut-off value: women < 80 cm, men < 94 cm for healthy measurement, women ≥ 80 cm, men ≥ 94 cm for elevated risk.

**Table 2:** Mean daily fibre intake and sources of intake from the Airwave Health Monitoring Study (*n* = 6898).

Achieving ≥30 g/day n (%)	231	3.3
	Mean	SD
**Total fibre g/day AOAC**	17.3	6.0
**Source of fibre by food group**		
**%non-whole grain cereal sources**	39.9	12.7
**% veg (exc. legume)**	16.2	8.5
**% potato (inc. crisps)**	13.6	8.6
**% whole grain**	12.4	9.8
**% fruit (exc. juice)**	11.2	9.2
**% legumes (inc. peanuts)**	6.0	5.7
**% nuts and seeds**	0.7	2.0

Abbreviations: AOAC Association of Analytical Chemists, IQR: NSP non-starch polysaccharides; SD standard deviation.

**Table 3:** Estimated mean body mass index, body fat percentage, waist circumference and C-reactive protein by quintile of energy-adjusted total fiber intake (g/1000 kcal) of Airwave Health Monitoring Study participants (*n* = 6898) ^1^.

Outcome Measure/Fiber Food Source	Model	Quintiles of Fibre Intake g/1000 kcal										
		Q1		Q2		Q3		Q4		Q5		*p_trend_*
		Adjusted Means and 95% Confidence Interval (95%CI)										
**Body Mass Index, kg/m^2^**												
**Total fibre**	Crude	26.99	26.77, 27.21	26.73	26.53, 26.93	26.86	26.66, 27.06	26.52	26.32, 26.72	26.52	26.32, 26.72	0.0001
	Adjusted	27.25	26.88, 27.62	26.94	26.57, 27.31	26.97	26.60, 27.34	26.54	26.17, 26.91	26.32	25.93, 26.71	<0.0001
**Whole grain fibre**	Crude	26.94	26.74, 27.14	26.82	26.62, 27.02	26.81	26.61, 27.01	26.63	26.43, 26.83	26.32	26.12, 26.52	<0.0001
	Adjusted	26.91	26.54, 27.28	26.90	26.53, 27.27	26.93	26.56, 27.30	26.78	26.41, 27.15	26.39	26.02, 26.76	0.0002
**Cereal non-whole grain**	Crude	26.92	26.72, 27.12	26.76	26.56, 26.96	26.64	26.44, 26.84	26.78	26.58, 26.98	26.44	26.24, 26.64	0.0026
	Adjusted	26.95	26.58, 27.32	26.82	26.45, 27.19	26.74	26.37, 27.11	26.86	26.49, 27.23	26.50	26.13, 26.87	0.0095
**Fruit fibre**	Crude	27.36	27.16, 27.56	26.72	26.52, 26.92	26.68	26.48, 26.88	26.26	26.06, 26.46	26.55	26.35, 26.75	<0.0001
	Adjusted	27.30	26.93, 27.67	26.79	26.42, 27.16	26.80	26.43, 27.17	26.38	26.01, 26.75	26.59	26.20, 26.98	<0.0001
**Vegetable and legume fibre**	Crude	26.90	26.68, 27.12	26.81	26.61, 27.01	26.44	26.24, 26.64	26.63	26.43, 26.83	26.76	26.56, 26.96	0.36
	Adjusted	27.02	26.65, 27.39	26.88	26.51, 27.25	26.54	26.17, 26.91	26.74	26.37, 27.11	26.70	26.33, 27.07	0.05
**Potato fibre**	Crude	26.56	26.36, 26.76	26.59	26.39, 26.79	26.53	26.33, 26.73	26.66	26.46, 26.86	27.19	26.99, 27.39	<0.0001
	Adjusted	26.85	26.48, 27.22	26.81	26.44, 27.18	26.64	26.27, 27.01	26.58	26.21, 26.95	26.92	26.53, 27.31	0.93
**Percentage body fat, %**												
**Total fibre**	Crude	28.13	27.80, 28.46	27.56	27.23, 27.89	27.49	27.16, 27.82	26.92	26.59, 27.25	26.80	26.47, 27.13	<0.0001
	Adjusted	28.10	27.51, 28.69	27.70	27.11, 28.29	27.57	26.98, 28.16	27.00	26.41, 27.59	26.77	26.16, 27.38	<0.0001
**Whole grain fibre**	Crude	28.06	27.73, 28.39	27.63	27.30, 27.96	27.43	27.1, 27.76	27.13	26.80, 27.46	26.58	26.25, 26.91	<0.0001
	Adjusted	27.59	27.00, 28.18	27.58	26.99, 28.17	27.50	26.91, 28.09	27.44	26.85, 28.03	26.81	26.22, 27.40	0.0007
**Cereal non-whole grain**	Crude	27.81	27.48, 28.14	27.37	27.04, 27.70	27.48	27.15, 27.81	27.26	26.93, 27.59	26.91	26.58, 27.24	0.0002
	Adjusted	27.58	26.99, 28.17	27.58	26.99, 28.17	27.56	26.97, 28.15	27.36	26.77, 27.95	27.36	26.77, 27.95	0.09
**Fruit fibre**	Crude	28.66	28.33, 28.99	27.68	27.35, 28.01	27.42	27.09, 27.75	26.54	26.21, 26.87	26.62	26.29, 26.95	<0.0001
	Adjusted	28.09	27.50, 28.68	27.54	26.95, 28.13	27.55	26.96, 28.14	26.86	26.27, 27.45	26.86	26.25, 27.47	<0.0001
**Vegetable and legume fibre**	Crude	27.71	27.38, 28.04	27.56	27.23, 27.89	26.93	26.6, 27.260	27.24	26.91, 27.57	27.41	27.08, 27.74	0.22
	Adjusted	27.66	27.07, 28.25	27.52	26.93, 28.11	27.03	26.44, 27.62	27.33	26.74, 27.92	27.33	26.74, 27.92	0.21
**Potato fibre**	Crude	26.58	26.25, 26.91	27.18	26.85, 27.51	27.33	27.00, 27.66	27.32	26.99, 27.65	28.40	28.07, 28.73	<0.0001
	Adjusted	27.18	26.59, 27.77	27.49	26.90, 28.08	27.40	26.81, 27.99	27.01	26.42, 27.60	27.65	27.04, 28.26	0.28
**Waist circumference, cm**												
**Total fibre**	Crude	88.75	88.24, 89.26	87.86	87.35, 88.37	87.94	87.43, 88.45	87.02	86.51, 87.53	86.55	86.04, 87.06	<0.0001
	Adjusted	89.04	88.10, 89.98	88.21	87.27, 89.15	88.16	87.22, 89.10	87.24	86.30, 88.18	86.57	85.59, 87.55	<0.0001
**Whole grain fibre**	Crude	88.43	87.92, 88.94	87.98	87.47, 88.49	87.80	87.29, 88.31	87.52	87.01, 88.03	86.25	85.74, 86.76	<0.0001
	Adjusted	88.08	87.16, 89.00	88.05	87.11, 88.99	88.02	87.10, 88.94	88.01	87.07, 88.95	86.73	85.79, 87.67	0.0004
**Cereal non-whole grain**	Crude	88.21	87.70, 88.72	87.66	87.15, 88.17	87.47	86.96, 87.98	87.84	87.33, 88.35	86.81	86.30, 87.32	0.0007
	Adjusted	88.09	87.15, 89.03	87.70	86.78, 88.62	87.69	86.75, 88.63	88.06	87.12, 89.00	87.21	86.25, 88.17	0.07
**Fruit fibre**	Crude	89.36	88.85, 89.87	88.16	87.65, 88.67	87.69	87.18, 88.20	86.43	85.92, 86.94	86.48	85.97, 86.99	<0.0001
	Adjusted	88.94	88.02, 89.86	88.16	87.24, 89.08	87.98	87.06, 88.90	86.84	85.90, 87.78	86.95	85.99, 87.91	<0.0001
**Vegetable and legume fibre**	Crude	88.34	87.81, 88.87	87.99	87.48, 88.50	87.10	86.59, 87.61	87.23	86.72, 87.74	87.41	86.90, 87.92	0.0083
	Adjusted	88.42	87.50, 89.34	88.01	87.09, 88.93	87.31	86.37, 88.25	87.53	86.59, 88.47	87.47	86.53, 88.41	0.0156
**Potato fibre**	Crude	86.69	86.18, 87.20	87.52	87.01, 88.03	87.21	86.70, 87.72	87.52	87.01, 88.03	89.01	88.50, 89.52	<0.0001
	Adjusted	87.56	86.62, 88.50	88.02	87.08, 88.96	87.44	86.50, 88.38	87.24	86.30, 88.18	88.40	87.44, 89.36	0.18
**C-reactive protein, mg/L**												
**Total fibre**	Crude	1.08	1.02, 1.14	1.01	0.96, 1.06	0.99	0.94, 1.04	0.90	0.85, 0.94	0.82	0.78, 0.86	<0.0001
	Adjusted	1.12	1.02, 1.23	1.08	0.98, 1.18	1.07	0.97, 1.17	0.97	0.88, 1.07	0.90	0.81, 0.99	<0.0001
**Whole grain fibre**	Crude	1.05	1.00, 1.11	0.98	0.93, 1.04	0.94	0.89, 0.99	0.91	0.87, 0.96	0.87	0.83, 0.92	<0.0001
	Adjusted	1.05	0.95, 1.15	1.03	0.94, 1.14	1.01	0.92, 1.11	1.01	0.92, 1.11	0.98	0.89, 1.08	0.10
**Cereal non-whole grain**	Crude	0.92	0.93, 1.03	0.97	0.92, 1.02	0.96	0.92, 1.01	0.94	0.89, 0.98	0.90	0.86, 0.95	0.0120
	Adjusted	1.02	0.92, 1.12	1.02	0.93, 1.12	1.04	0.95, 1.15	1.01	0.92, 1.11	1.00	0.91, 1.10	0.64
**Fruit fibre**	Crude	1.12	1.06, 1.18	1.03	0.98, 1.09	0.97	0.92, 1.02	0.84	0.80, 0.89	0.83	0.79, 0.88	<0.0001
	Adjusted	1.11	1.01, 1.22	1.07	0.98, 1.18	1.05	0.95, 1.15	0.93	0.85, 1.02	0.95	0.86, 1.05	<0.0001
**Vegetable and legume fibre**	Crude	1.03	0.97, 1.08	1.05	1.00, 1.10	0.91	0.87, 0.96	0.90	0.86, 0.95	0.89	0.85, 0.94	<0.0001
	Adjusted	1.07	0.97, 1.17	1.11	1.01, 1.22	0.98	0.89, 1.08	0.97	0.88, 1.07	0.97	0.88, 1.06	0.0005
**Potato fibre**	Crude	0.86	0.82, 0.91	0.92	0.88, 0.97	0.96	0.91, 1.01	0.95	0.90, 1.00	1.07	1.02, 1.13	<0.0001
	Adjusted	0.98	0.89, 1.08	1.02	0.93, 1.12	1.02	0.93, 1.13	0.98	0.89, 1.08	1.06	0.96, 1.17	0.15

^1^*n* = 6898 due to missing covariates in adjusted models. General linear models were used to obtain adjusted means and 95% confidence intervals, orthogonal polynomial coefficients derived from median intake in each quintile were applied to estimate p-trend across quintiles of intake. *n* = 6714 due to missing covariates. Crude: Adjusted for age and sex. Adjusted model additionally adjusted for age and sex, ethnic category, marital status, final attained educational level, length or weekly working hours, smoking (current, previous, never), daily TV viewing hours and physical activity (IPAQ category) and continuous variable: alcohol intake (median g/day), continuous variables of mean daily intakes of: total energy, energy from saturated fat, polyunsaturated fat, non-milk extrinsic sugars (continuous variables), other sources fibre than one under study (continuous variables) and potato fibre additionally adjusted for fried potato consumption (categorical: non-consumers, low and high). C-reactive protein log transformed for analyses, back transformed values presented. Quintile ranges (g/1000 kcal). Total fibre Q: 2.53–6.85, Q2: 6.86–8.17, Q3: 8.18–9.48, Q4: 9.49–11.25, Q5 11.26–32.98. Whole grain fibre: Q1: 0.00–0.20, Q2: 0.21–0.70, Q3: 0.71–1.3, Q4: 1.31–2.09, Q5: 2.10–10.77. Cereal non-whole grain fibre: Q1: 0.15–2.65, Q2: 2.66–3.17, Q3: 3.18–3.65, Q4: 3.66–4.36, Q5: 4.37–14.48. Fruit fibre: Q1: 0.00–0.20, Q”: 0.21–0.61, Q3: 0.62–1.08, Q4: 1.08–1.83, Q5: 1.84–11.02. Vegetable and legume fibre: Q1: 0.00–1.03, Q2: 1.04–1.54, Q3: 1.55–2.11, Q4: 2.12–2.94, Q5’: 2.95–14.85. Potato fibre: Q1: 0.00–0.57, Q2: 0.58–0.90, Q3: 0.91–1.22, Q4: 1.23–1.68, Q5: 1.69–5.97.

## Supplementary Materials

The following are available online at https://www.mdpi.com/2072-6643/11/8/1839/s1. Figure S1: Airwave Health Monitoring Study participant flow chart for inclusion in the cross-sectional study: The association between food sources of dietary fibre with measures of body composition and inflammation; Table S1: Food group descriptions applied to the dietary data from the Airwave Health Monitoring Study; Table S2: Cut-off values per quintile group of dietary fibre intake (energy-adjusted); Table S3: Characteristics of Airwave Health Monitoring Study participants by quintile of energy-adjusted dietary fibre intakes; Table S4: The association between fibre intakes and measures of body composition and inflammation - estimated beta coefficients. Tables S5 and S6: Analyses stratified by body mass index category; Tables S7 and S8: Analyses stratified by carbohydrate intake; Table S9 and S10: Analyses stratified by classification of energy intake reporting

## References

[1] Kelly T., Yang W., Chen C.S., Reynolds K., He J. (2008). Global burden of obesity in 2005 and projections to 2030. Int. J. Obes..

[2] McKinsey Global Institute (2014). Overcoming Obesity: An Initial Economic Analysis. www.mckinsey.com/mgi.

[3] Hill J.O., Commerford R. (1996). Physical activity, fat balance, and energy balance. Int. J. Sport Nutr..

[4] Mozaffarian D., Hao T., Rimm E.B., Willett W.C., Hu F.B. (2011). Changes in Diet and Lifestyle and Long-Term Weight Gain in Women and Men. N. Engl. J. Med..

[5] Sperling L.S., Mechanick J.I., Neeland I.J., Herrick C.J., Despres J.P., Ndumele C.E., Blum Q.K. (2015). The CardioMetabolic Health Alliance: Working Toward a New Care Model for the Metabolic Syndrome. J. Am. Coll. Cardiol..

[6] Alfieri M.A.H., Pomerleau J., Grace D.M., Anderson L. (1995). Fiber Intake of Normal Weight, Moderately Obese and Severely Obese Subjects. Obes. Res..

[7] Murphy N., Norat T., Ferrari P., Jenab M., Bueno-de-Mesquita B., Skeie G., Clavel-Chapelon F. (2012). Dietary Fibre Intake and Risks of Cancers of the Colon and Rectum in the European Prospective Investigation into Cancer and Nutrition (EPIC). PLoS ONE.

[8] InterAct Consortium TI (2015). Dietary fibre and incidence of type 2 diabetes in eight European countries: The EPIC-InterAct Study and a meta-analysis of prospective studies. Diabetologia.

[9] Van de Vijver L.P., van den Bosch L.M., van den Brandt P.A., Goldbohm R.A. (2009). Whole-grain consumption, dietary fibre intake and body mass index in the Netherlands cohort study. Eur. J. Clin. Nutr..

[10] Du H., van der A.D.L., Boshuizen H.C., Forouhi N.G., Wareham N.J., Halkjaer J. (2010). Dietary fiber and subsequent changes in body weight and waist circumference in European men and women. Am. J. Clin. Nutr..

[11] AlEssa H.B., Cohen R., Malik V.S., Adebamowo S.N., Rimm E.B., Manson J.E. (2018). Carbohydrate quality and quantity and risk of coronary heart disease among US women and men. Am. J. Clin. Nutr..

[12] Bingham S.A., Gill C., Welch A., Day K., Cassidy A., Khaw K.T., Day N.E. (1994). Comparison of dietary assessment methods in nutritional epidemiology: Weighed records v. 24 h recalls, food-frequency questionnaires and estimated-diet records. Br. J. Nutr..

[13] Lapice E., Maione S., Patti L., Cipriano P., Rivellese A.A., Riccardi G., Vaccaro O. (2009). Abdominal adiposity is associated with elevated C-reactive protein independent of BMI in healthy nonobese people. Diabetes Care.

[14] Eckel R.H., Cornier M.-A. (2014). Update on the NCEP ATP-III emerging cardiometabolic risk factors. BMC Med..

[15] Libby P., Ridker P.M., Hansson G.K. (2009). Inflammation in atherosclerosis: From pathophysiology to practice. J. Am. Coll. Cardiol..

[16] Elliott P., Vergnaud A.C., Singh D., Neasham D., Spear J., Heard A. (2014). The Airwave Health Monitoring Study of police officers and staff in Great Britain: Rationale, design and methods. Environ. Res..

[17] Gibson R., Eriksen R., Lamb K., McMeel Y., Vergnaud A.C., Spear J., Frost G. (2017). Dietary assessment of British police force employees: A description of diet record coding procedures and cross-sectional evaluation of dietary energy intake reporting (The Airwave Health Monitoring Study). BMJ Open.

[18] Willett W., Howe G., Kushi L. (1997). Adjustment for total energy intake in epidemiologic studies. Am. J. Clin. Nutr..

[19] Black A.E. (2000). Critical evaluation of energy intake using the Goldberg cut-off for energy intake:basal metabolic rate. A practical guide to its calculation, use and limitations. Int. J. Obes. Relat. Metab. Disord..

[20] Jones A.R., Mann K.D., Kuznesof S.A., Richardson D.P., Seal C.J. (2017). The whole grain content of foods consumed in the UK. Food Chem..

[21] Kang M.Y., Park H., Seo J.C., Kim D., Lim Y.H., Lim S., Hong Y.C. (2012). Long working hours and cardiovascular disease: A meta-analysis of epidemiologic studies. J. Occup. Environ. Med..

[22] Kivimäki M., Virtanen M., Kawachi I., Nyberg S.T., Alfredsson L., Batty G.D., Dragano N. (2014). Long working hours, socioeconomic status, and the risk of incident type 2 diabetes: A meta-analysis of published and unpublished data from 222 120 individuals. Lancet Diabetes Endocrinol..

[23] Craig C.L., Marshall A.L., Sjöström M., Bauman A.E., Booth M.L., Ainsworth B.E. (2003). International physical activity questionnaire: 12-country reliability and validity. Med. Sci. Sports Exerc..

[24] (2005). IPAQ Scoring Protocol. http://www.ipaq.ki.se/scoring.htm.

[25] Coulombe D. Orthogonal polynomial coefficients and trend analysis for unequal intervals and unequal Ns: A microcomputer application. Behav. Res. Methods 1985..

[26] Muraki I., Rimm E.B., Willett W.C., Manson J.E., Hu F.B., Sun Q. (2016). Potato Consumption and Risk of Type 2 Diabetes: Results from Three Prospective Cohort Studies. Diabetes Care.

[27] Borch D., Juul-Hindsgaul N., Veller M., Astrup A., Jaskolowski J., Raben A. (2016). Potatoes and risk of obesity, type 2 diabetes, and cardiovascular disease in apparently healthy adults: A systematic review of clinical intervention and observational studies. Am. J. Clin. Nutr..

[28] Tooze J.A., Freedman L.S., Carroll R.J., Midthune D., Kipnis V. (2016). The impact of stratification by implausible energy reporting status on estimates of diet-health relationships. Biom. J..

[29] Ma Y., Griffith J.A., Chasan-Taber L., Olendzki B.C., Jackson E., Stanek E.J. (2006). Association between dietary fiber and serum C-reactive protein. Am. J. Clin. Nutr..

[30] Threapleton D., Greenwood D.C., Evans C.E.L., Cleghorn C.L., Nykjaer C., Woodhead C. (2013). Dietary fibre intake and risk of cardiovascular disease: Systematic review and meta-analysis. BMJ.

[31] Ma Y., Griffith J.A., Chasan-Taber L., Olendzki B.C., Jackson E., Stanek III E.J., Ockene I.S. (2017). Association In Proceedings of the and dietary food sources of fibre in Spain: Differences with regard to the prevalence of excess body weight and abdominal obesity in adults of the ANIBES study. Nutrients.

[32] Borgi L., Rimm E.B., Willett W.C., Forman J.P. (2016). Potato intake and incidence of hypertension: Results from three prospective US cohort studies. BMJ.

[33] Birt D.F., Boylston T., Hendrich S., Jane J.L., Hollis J., Li L., Schalinske K. (2013). Resistant starch: Promise for improving human health. Adv. Nutr..

[34] Parnell J.A., Reimer R.A. (2009). Weight loss during oligofructose supplementation is associated with decreased ghrelin and increased peptide YY in overweight and obese adults. Am. J. Clin. Nutr..

[35] Cani P.D., Joly E., Horsmans Y., Delzenne N.M. (2006). Oligofructose promotes satiety in healthy human: A pilot study. Eur. J. Clin. Nutr..

[36] Moshfegh A.J., Friday J.E., Goldman J.P., Ahuja J.K. (1999). Presence of inulin and oligofructose in the diets of Americans. J. Nutr..

[37] Edwards C.A., Havlik J., Cong W., Mullen W., Preston T., Morrison D.J., Combet E. (2017). Polyphenols and health: Interactions between fibre, plant polyphenols and the gut microbiota. Nutr. Bull..

[38] Chambers E.S., Viardot A., Psichas A., Morrison D.J., Murphy K.G., Zac-Varghese S.E., Blundell J.E. (2015). Effects of targeted delivery of propionate to the human colon on appetite regulation, body weight maintenance and adiposity in overweight adults. Gut.

[39] Whitton C., Nicholson S.K., Roberts C., Prynne C.J., Pot G.K., Olson A., Henderson H. (2014). NDNS Survey Results from Years 7 and 8 of the Rolling Programme. www.gov.uk/phe.

[40] Gibson R., Eriksen R., Singh D., Vergnaud A.C., Heard A., Chan Q. (2017). A cross-sectional investigation into the occupational and socio-demographic characteristics of British police force employees reporting a dietary pattern associated with cardiometabolic risk: Findings from the Airwave Health Monitoring Study. Eur. J. Nutr..

[41] Mann K.D., Pearce M.S., Seal C.J. (2017). Providing evidence to support the development of whole grain dietary recommendations in the United Kingdom. Proc. Nutr. Soc..

[42] Kromhout D., Spaaij C.J.K., de Goede J., Weggemans R.M. (2016). The 2015 Dutch food-based dietary guidelines. Eur. J. Clin. Nutr..

[43] Alinia S., Hels O., Tetens I. (2009). The potential association between fruit intake and body weight—A review. Obes Rev..

[44] Williamson D.F., Kahn H.S., Remington P.L., Anda R.F. (1990). The 10-year incidence of overweight and major weight gain in US adults. Arch. Intern. Med..

[45] (2014). Cohort Strategic Review Subgroup. Medical Research Council. Maximising the Value of UK Population Cohorts. MRC Strategic Review of the Largest UK Population Cohort Studies. https://mrc.ukri.org/publications/browse/maximising-the-value-of-uk-population-cohorts/.

[46] Lafay L., Mennen L., Basdevant A., Charles M.A., Borys J.M., Eschwege E., Romon M. (2000). Does energy intake underreporting involve all kinds of food or only specific food items? Results from the Fleurbaix Laventie Ville Santé (FLVS) study. Int. J. Obes. Relat. Metab. Disord..

