# Supplementary material for: Intakes and Food Sources of Dietary Fibre and Their Associations with Measures of Body Composition and Inflammation in UK Adults: Cross-Sectional Analysis of the Airwave Health Monitoring Study
Source: Nutrients. 2019 Aug 8;11(8):1839. doi: 10.3390/nu11081839 (PMC6722677; doi:10.3390/nu11081839)
Supplement: Supplementary file 1 [file nutrients-11-01839-s001.pdf]

Online Supplementary Material

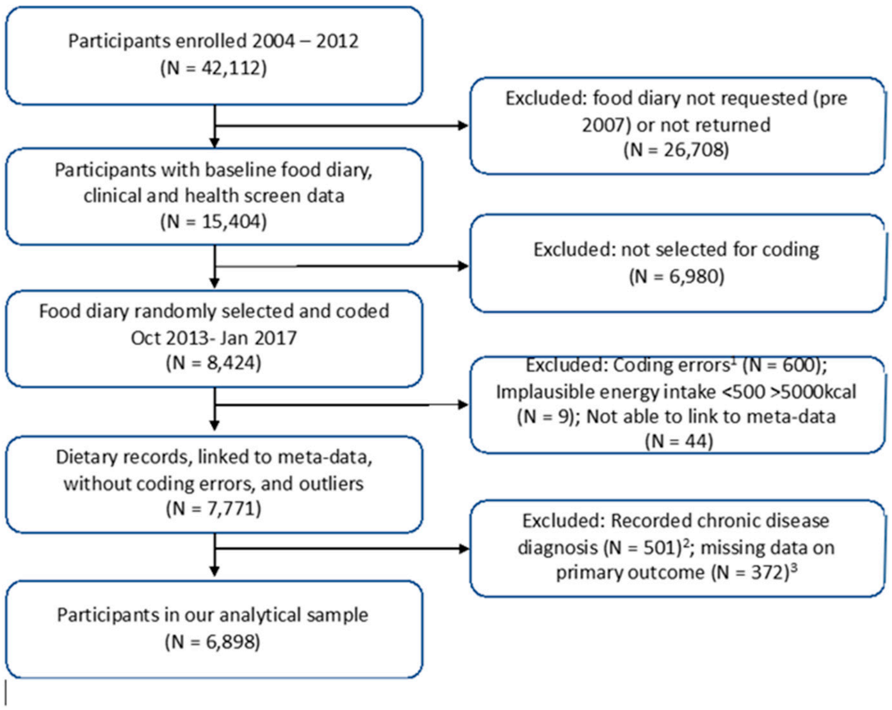

Figure S 1 Airwave Health Monitoring Study participant flow chart for inclusion in the cross-sectional study: The association between food sources of dietary fibre with measures of body composition and inflammation.

[1] Refer to dietary coding protocol available at: https://bmjopen.bmj.com/content/7/4/e012927
[2] Self-report General Practitioner diagnoses of: angina, heart disease, chronic obstructive pulmonary disease, cancer, chronic liver disease, thyroid disease, arthritis, diabetes (type 1 or diagnosed type 2), previous stroke
[3] Missing C-Reactive protein and/or body fat measurement (no participants had missing BMI or waist circumference data)

Table S1 Food group descriptions applied to the dietary data from the Airwave Health Monitoring Study

Food group	Items included	Items not included
Whole grains*	Amaranth<br>Barley, whole, Barley meal<br>Brown rice, Brown rice flour, Wild rice<br>Buckwheat<br>Bulgur wheat<br>Millett<br>Oats, Oat flour, Oatmeal<br>Popcorn (only include plain)<br>Quinoa<br>Whole wheat flour<br>Shredded wheat<br>Dark rye bread / pumpernickel<br>Muesli<br>Ready Brek / quick cook oats<br>Weetabix, Cheerios, Shreddies, All-bran<br>Whole wheat pasta<br>Ryvita / rye crackers<br>Oatcakes<br>Whole wheat bread<br>Flapjack (% oats)	Barley, pearled<br>Barley malt flour<br>Bran (all grains)<br>Corn flour/meal<br>Couscous<br>Rice (milled, not whole grain)<br>Semolina<br>Wheat flour (milled, not whole grain)<br>Brown flour<br>Brown bread<br>Cornflakes<br>Rice krispie<br>Egg / rice / plain noodles<br>Nutri-grain<br>Granary bread made with white flour
Cereal (non-wholegrain sources)	All cereal products – bread, flour, breakfast cereal, rice, crackers, crisp breads.<br>Enriched dough breads – teacakes. Scones. Crumpets. English muffins.<br>Cakes, pastry, biscuits made with cereal flour,<br>Biscuits sweet, sweet pastries and cakes, puddings (sponge, tarts), croissants, Danish pastry, (cereal / dairy based)	
Legume	Runner, broad beans<br>Garden peas<br>Snow peas (mangetout)<br>Beans<br>Lentils<br>Pea nuts<br>Chickpeas	Processed soya bean products<br>Bean flours<br>French beans, string beans, snap beans and sugar snaps
Potato	All methods of cooking<br>Chips (friend and oven)<br>Mashed, boiled, crisps<br>Inc. as part of composite dish – e.g. topping on shepherd’s pie	Potato flour/starch as added to manufactured foods

Fried / cooked in oil potato	Potato crisps (not baked)<br>Fried chips, roasted in oil (all oils)	
Nuts, seeds	Seeds used in seasoning (fennel, cumin, caraway)<br>Sunflower<br>Pumpkin<br>Thaini<br>Pine nut (= seed)	Seed oils<br>Peanuts (ground nuts)<br>Seeds in fruit<br>Hemp milk<br>Nut milks<br>Coconut<br>Marzipan and as part of confectionary
Fruit (UK culinary usage classification)	Fresh fruit<br>Dried fruit<br>Pre-prepared fruit including frozen and canned)<br>100% pure fruit juices and smoothies	Fruit in cakes and confectionary<br>Jam / chutney<br>Fruit juices as cordial
Vegetables (UK culinary usage classification)	Sweet potato<br>All fresh, and prepared vegetables (frozen, dried, canned). Seaweeds<br>Herbs - fresh<br>Tomatoes	White potato<br>Cassava<br>Chutney, ketchup<br>Dried herbs

***% whole grain content based on manufactures and published declarations**

Table S2 Cut-off values per quintile group of dietary fibre intake (energy adjusted)

	20%	40%	60%	80%
Total g/day per 1000 kcal	6.85	8.17	9.48	11.25
Wholegrain g/day per 1000kcal	0.20	0.70	1.30	2.09
Legume g/day per 1000kcal	0.08	0.30	0.53	0.93
Fruit g/day (exc. juices) per 1000kcal	0.20	0.62	1.09	1.83
Vegetable g/day (exc. potato) per 1000kcal	0.73	1.11	1.53	2.14
Potato g/day per 1000kcal	0.58	0.90	1.22	1.68
Non-wholegrain cereal g/day per 1000kcal	2.65	3.17	3.66	4.36

Table S3 Characteristics of Airwave Health Monitoring Study participants by quintile of energy-adjusted dietary fibre intakes (g/1000kcal) *N* = 6 898

	Quintile of dietary fibre intake (g/1000kcal)										
	**Q1**		**Q2**		**Q3**		**Q4**		**Q5**		**P#**
N	1379		1380		1380		1380		1379		
Fibre intake g/1000kcal, mean, SD	5.8	0.8	7.5	0.4	8.8	0.4	10.3	0.5	13.3	2.0	
**Men N (%)**	952	69.0	864	62.6	876	63.5	810	58.7	781	52.1	<0.0001
**Age, years (SD)**	39.3	9.1	40.3	9.0	41.5	8.8	41.9	9.0	42.6	9.2	
**White N (%)**	1324	96.1	1340	97.2	1344	97.46	1350	97.9	1349	97.8	0.0236
**Relationship status**											0.06
Cohabiting	249	18.7	234	17.4	218	16.1	217	16.1	207	15.3	
Divorced/separated	111	8.4	100	7.4	114	8.4	108	8	107	7.9	
Married	799	60.2	860	63.7	893	65.8	890	64.8	866	64.1	
Single	169	12.7	155	11.5	132	9.7	137	10.3	170	12.6	
Missing	*51*		*31*		*23*		*28*		*29*		
**Education**											0.0135
Left school before taking GCSE	56	4.1	41	3	42	3	54	3.9	66	4.8	
GCSE or equivalent	420	30.5	419	30.4	385	27.9	418	30.3	407	29.5	
Vocational qualifications	114	8.3	119	8.6	93	6.7	87	6.3	85	6.2	
A-levels / Highers or equivalent	460	33.4	437	31.7	450	32.6	444	32.2	427	31	
Bachelor's degree or equivalent	261	18.9	287	20.8	310	22.5	279	20	305	22.1	
Postgraduate qualifications	68	4.9	76	5.5	100	7.25	98	7.1	89	6.5	
**Employment force, country**											<0.0001
England	958	69.7	1006	73	1035	75.2	1072	77.7	1104	80.3	
Scotland	310	22.6	261	18.9	217	15.8	175	12.7	130	9.4	
Wales	106	7.7	111	8.1	124	9	132	9.6	141	10.2	
Missing	*5*		*2*		*4*		*1*		*4*		
**Rank**											<0.0001
Police Constable/ Sergeant	74	6.3	77	6.7	102	9.2	86	7.9	94	8.5	
Inspector and above	775	66.3	713	62.2	636	57.2	600	55.2	571	51.5	
Police staff / Other	320	27.4	356	31.1	373	33.6	402	39.9	444	40.0	
Missing	*210*		*234*		*269*		*292*		*270*		

**Hours worked per week**											<0.0001
<35	68	4.9	114	8.3	131	9.5	139	10.1	144	10.4	
35 <40	477	34.6	512	37.1	513	37.2	542	39.3	593	43.0	
40 <49	511	37.1	466	33.8	452	32.8	430	31.2	401	29.1	
49 <55	165	12.0	153	10.4	151	10.9	127	9.2	140	10.2	
55+	158	11.5	145	10.5	133	9.6	142	10.3	101	7.3	
**Physical activity (MET category)**											<0.0001
Low	188	13.6	159	11.5	163	11.8	130	9.4	132	9.6	
Moderate	687	49.8	650	47.1	619	44.9	582	42.2	549	39.8	
High	504	36.6	571	41.4	598	43.3	668	48.4	698	50.6	
**Smoking status**											<0.0001
Never smoker	890	64.8	946	68.8	977	71.1	971	70.6	996	72.4	
Former smoker	307	22.3	299	21.8	293	21.3	324	23.6	319	23.2	
Current smoker	177	12.9	130	9.5	105	7.6	81	5.9	61	4.4	
Missing	*5*		*5*		*5*		*4*		*3*		
**Weekly TV viewing**											0.0022
Low (< 6 hours)	363	26.3	421	30.5	410	29.7	416	30.1	438	31.8	
Moderate (6 – 15 hours)	622	45.1	584	42.3	605	43.8	629	45.6	635	46.1	
High (>15 hours)	394	28.6	375	27.2	365	26.4	335	24.3	306	22.2	
**Body mass index category**											0.0004
Healthy (<25kg/m2)	397	28.8	471	34.1	437	31.7	487	32.3	511	37.1	
Overweight (>25 <30kgm^2)	702	50.9	651	47.2	658	47.7	649	47.0	628	45.5	
Obese (>30kg/m^2)	280	20.3	258	18.7	285	20.6	244	17.7	240	17.4	
**Source fibre: mean, SD**											
% whole grains	7.19	8.37	9.66	8.45	12.84	9.20	15.02	9.45	17.37	9.90	<0.0001
% fruit (exc. juice)	6.73	7.34	9.38	8.07	11.04	8.35	13.23	9.41	15.81	9.86	<0.0001
% veg (exc. Legumes and potato)	14.42	8.92	15.68	7.82	16.25	7.87	16.65	8.41	17.92	9.12	<0.0001
% legume	4.46	5.06	5.67	5.57	6.00	5.37	6.23	5.67	7.44	6.25	<0.0001
% nuts and seeds	0.45	1.51	0.53	1.44	0.68	1.54	0.81	2.04	0.99	2.95	<0.0001
% potatoes	13.83	9.19	11.79	7.21	10.69	6.66	9.58	6.45	8.15	5.98	<0.0001
% non-wholegrain cereal sources	48.76	12.71	43.82	10.88	39.71	10.40	36.36	10.54	30.95	11.17	<0.0001

**Nutrient intakes, mean SD**											
Energy, mean kcal/day	2047.6	508.6	1991.6	500.4	1947.2	457.7	1868.7	467.3	1761.3	462.9	<0.0001
% energy intake – total fat	35.45	5.42	34.89	5.20	34.09	4.94	32.69	4.96	30.24	5.39	<0.0001
% energy intake – saturated fat	13.43	2.90	13.03	2.73	12.48	2.58	11.84	2.58	10.51	2.60	<0.0001
% energy intake – PUFA	5.72	1.53	5.83	1.62	5.85	1.59	5.67	1.52	5.59	1.71	<0.0001
% energy intake - carbohydrate	43.78	7.41	45.72	6.39	46.85	6.12	48.55	6.01	51.00	6.32	<0.0001
% energy intake NME	13.32	5.80	12.19	5.03	11.81	4.64	11.37	4.26	10.23	4.34	<0.0001
% energy intake - protein	16.53	3.84	16.72	3.09	16.96	3.00	17.34	3.27	18.17	3.60	<0.0001
**Median IQR**											
% energy intake – Alcohol*	6.7	9.05	5.42	7.18	5.00	6.46	4.59	5.84	3.9	5.49	<0.0001
Vegetables grams/1000kcal	42.7	31.70	57.35	38.95	68.08	42.93	78.00	52.90	100.4	70.83	<0.0001
Fruit grams/1000kcal exc juice	14.0	33.16	31.87	43.01	44.85	52.90	49.34	61.30	105.2	70.89	<0.0001
Wholegrain grams/1000kcal	4.6	9.69	8.98	11.95	14.58	14.43	19.76	17.36	28.4	21.96	<0.0001
Potato and products g/1000kcal	31.9	27.74	35.36	28.78	37.54	31.94	38.94	31.82	41.8	39.19	<0.0001
Legumes (inc. peanut) g/1000kcal	5.1	9.14	8.14	10.75	10.33	12.04	12.21	14.19	17.5	18.24	<0.0001
Total dairy grams/1000kcal	98.0	78.37	110.73	78.90	120.33	81.62	133.09	86.29	140.8	100.41	<0.0001
Fish grams/1000kcal	7.0	15.40	8.87	18.00	9.29	16.88	11.54	17.93	13.7	21.59	<0.0001
Total red and processed meat g/1000kcal	21.2	18.91	20.71	14.12	18.57	13.04	17.38	13.18	14.7	13.46	<0.0001
Sugar sweetened drinks g/1000kcal	54.4	150.9	34.12	116.5	28.98	94.63	18.69	76.57	0.0	45.00	<0.0001
Sodium mg/1000kcal	384.5	636.4	366.4	683.0	372.5	688.0	411.9	517.2	459.1	389.7	0.0245

#Association between categorical variables tested by Chi-squared analysis (missing values excluded), across normal distributed data (mean SD) general linear models were applied, across non-normal distributed data (median IQR) Kruskal-Wallis test. *consumers of alcohol only. Abbreviations: BIA bio electrical impedance, CRP C-reactive protein, GCSE general certificate secondary education, IQR interquartile range, MET metabolic equivalents, PUFA poly unsaturated fatty acids, NME non-milk extrinsic sugars, SD standard deviation.

Table S4 The association between fibre intakes and measures of body composition and inflammation - estimated beta coefficients (95% confidence intervals).

			95% Confidence intervals		
		Beta coefficient	lower	upper	P
**Body Mass Index, kg/m2**					
Total fibre	Crude	-0.07	-0.10	-0.03	<0.0001
	Adjusted	-0.13	-0.17	-0.09	<0.0001
Wholegrain fibre*	Crude	-0.45	-0.63	-0.26	<0.0001
	Adjusted	-0.40	-0.60	-0.20	<0.0001
Cereal non-whole grain*	Crude	-0.41	-0.72	-0.11	0.008
	Adjusted	-0.40	-0.73	-0.07	0.018
Fruit fibre*	Crude	-0.60	-0.77	-0.42	<0.0001
	Adjusted	-0.57	-0.77	-0.38	<0.0001
Vegetable and legume fibre*	Crude	-0.15	-0.36	0.06	0.16
	Adjusted	-0.26	-0.48	-0.04	0.02
Potato fibre*	Crude	0.56	0.30	0.82	<0.0001
	Adjusted	0.05	-0.28	0.39	0.76
**Percentage body fat, %**					
Total fibre	Crude	-0.17	-0.23	-0.12	<0.0001
	Adjusted	-0.19	-0.26	-0.13	<0.0001
Wholegrain fibre*	Crude	-1.06	-1.3	-0.77	<0.0001
	Adjusted	-0.57	-0.88	-0.26	0.0003
Cereal non-whole grain*	Crude	-0.94	-1.43	-0.45	0.0002
	Adjusted	-0.46	-0.99	0.059	0.08
Fruit fibre*	Crude	-1.47	1.74	-1.19	<0.0001
	Adjusted	-0.96	-1.26	-0.64	<0.0001
Vegetable and legume fibre*	Crude	-0.27	-0.61	0.06	0.11
	Adjusted	-0.19	0.30	-0.54	0.17
Potato fibre*	Crude	1.63	1.21	2.05	<0.0001
	Adjusted	0.45	-0.07	0.98	0.09
**Waist circumference, cm**					
Total fibre	Crude	-0.28	-0.36	-0.19	<0.0001
	Adjusted	-0.34	-0.44	-0.23	<0.0001
Wholegrain fibre*	Crude	-1.52	-1.99	-1.05	<0.0001
	Adjusted	-0.95	-1.45	-0.46	0.0002
Cereal non-whole grain*	Crude	-1.08	-1.84	-0.32	0.006
	Adjusted	-0.51	-1.34	0.32	0.23
Fruit fibre*	Crude	-2.16	-2.60	-1.73	<0.0001
	Adjusted	-1.68	-2.17	-1.19	<0.0001
Vegetable and legume fibre*	Crude	-0.72	-1.24	-0.20	0.01
	Adjusted	-0.60	-1.16	-0.04	0.04
Potato fibre*	Crude	2.02	1.37	2.67	<0.0001

	Adjusted	0.73	-0.10	1.57	0.09
**C-reactive protein, mg/L**					
Total fibre	Crude	-0.03	-0.04	-0.02	<0.0001
	Adjusted	-0.03	-0.04	-0.02	<0.0001
Wholegrain fibre*	Crude	-0.13	-0.18	-0.09	<0.0001
	Adjusted	-0.04	-0.09	0.007	0.09
Cereal non-whole grain*	Crude	-0.10	-0.18	-0.02	0.011
	Adjusted	-0.02	-0.11	0.06	0.62
Fruit fibre*	Crude	-0.22	-0.26	-0.17	<0.0001
	Adjusted	-0.14	-0.18	-0.09	<0.0001
Vegetable and legume fibre*	Crude	-0.13	-0.18	-0.08	<0.0001
	Adjusted	-0.08	0.06	0.18	0.00
Potato fibre*	Crude	0.18	0.12	0.25	<0.0001
	Adjusted	0.06	-0.02	0.15	0.15

All fibre intakes reported in g/1000kcal. Total fibre regression coefficient represents change in outcome measure per 1g/1000kcal day increase in fibre. *Square root transformation applied to independent variable (fibre intake) for linear regression - regression coefficient change in outcome measure per square root (1g/1000kcal day) increase in fibre.
Crude: Adjusted for age and sex. Adjusted Model additionally adjusted for age and sex, ethnic category, marital status, final attained educational level, length or weekly working hours, smoking (current, previous, never), daily TV viewing hours and physical activity (IPAQ category) and continuous variable: alcohol intake (median g/day), continuous variables of mean daily intakes of: total energy, energy from saturated fat, polyunsaturated fat, non-milk extrinsic sugars (continuous variables), other sources fibre than one under study (continuous variables) and potato fibre additionally adjusted for fried potato consumption (categorical: non-consumers, low and high). C-reactive protein log transformed for analyses.

Table S5 and 6: Stratified by body mass index category

Table S5 Estimated mean waist circumference, C-reactive protein, and body fat percentage by quintile of energy-adjusted total fibre intake (g/1000kcal) of Airwave Health Monitoring Study participants with a healthy body mass index <25kg/m^2 (N = 2 303)

	Quintiles of energy adjusted total fibre intake (g/1000kcal)										
	Q1		Q2		Q3		Q4		Q5		P_{trend}
	Adjusted mean SE										
Waist circumference, cm											
Model 1	88.35	0.46	87.46	0.44	87.47	0.44	86.25	0.44	85.63	0.43	<0.0001
Model 2	88.52	0.80	87.61	0.80	87.36	0.80	86.27	0.81	85.26	0.83	<0.0001
C-Reactive protein, mg/L											
Model 1	1.07	1.04	0.96	1.04	0.97	1.04	0.83	1.04	0.75	1.04	<0.0001
Model 2	1.12	1.07	1.03	1.07	1.04	1.07	0.91	1.07	0.83	1.08	<0.0001
Body fat, %											
Model 1	27.54	0.29	26.74	0.27	26.87	0.27	25.85	0.27	25.85	0.27	<0.0001
Model 2	27.32	0.49	26.74	0.49	26.71	0.49	25.80	0.50	25.60	0.51	<0.0001

Adjusted means and standard error of the mean (SE) presented. Orthogonal polynomial coefficients derived from median intake in each quintile were applied to estimate p-trend across quintiles of intake using general linear models. Model 1: age, and sex. Model 2 adjusted for: age, sex ethnic category, marital status, final attained educational level, length or weekly working hours, smoking (current, previous, never), daily TV viewing hours and physical activity (IPAQ category) and continuous variable: alcohol intake (median g/day), continuous variables of mean daily intakes of: total energy, energy from saturated fat, polyunsaturated fat, non-milk extrinsic sugars (continuous variables). C-reactive protein log transformed for analyses.

Table S6 Estimated mean waist circumference, C-reactive protein, and body fat percentage by quintile of energy-adjusted total fibre intake (g/1000kcal) of Airwave Health Monitoring Study participants with a body mass index $\geq$ 25kg/m^2 (N = 4 595)

	Quintiles of energy adjusted total fibre intake (g/1000kcal)										
	Q1		Q2		Q3		Q4		Q5		P_{trend}
	Adjusted mean SE										
Waist circumference, cm											
Model 1	93.7	0.3	93.1	0.3	93.1	0.3	92.0	0.3	91.8	0.3	<0.0001
Model 2	93.8	0.5	93.2	0.5	93.3	0.5	92.2	0.5	91.9	0.6	<0.0001
C-Reactive protein, mg/L											
Model 1	1.33	1.03	1.32	1.03	1.23	1.03	1.14	1.03	1.07	1.03	<0.0001
Model 2	1.34	1.06	1.37	1.06	1.30	1.06	1.22	1.06	1.16	1.06	<0.0001
Body fat, %											
Model 1	31.27	0.17	31.12	0.17	30.92	0.17	30.51	0.17	30.44	0.17	<0.0001
Model 2	31.16	0.30	31.09	0.30	30.93	0.30	30.52	0.30	30.40	0.32	0.0012

Adjusted means and standard error of the mean (SE) presented. Orthogonal polynomial coefficients derived from median intake in each quintile were applied to estimate p-trend across quintiles of intake using general linear models. Model 1: age, and sex. Model 2 adjusted for: age, sex ethnic category, marital status, final attained educational level, length or weekly working hours, smoking (current, previous, never), daily TV viewing hours and physical activity (IPAQ category) and continuous variable: alcohol intake (median g/day), continuous variables of mean daily intakes of: total energy, energy from saturated fat, polyunsaturated fat, non-milk extrinsic sugars (continuous variables). C-reactive protein log transformed for analyses.

Table S 7 and S 8: Stratified by carbohydrate intake

Table S 7 Relationships between total fibre intake (g/1000kcal) and body mass index, waist circumference, C-reactive protein and body fat of Airwave Health Monitoring Study participants with high carbohydrate intake (≥50% EI carbohydrate) (N = 2 357)

	Quintiles of energy adjusted total fibre intake (g/1000kcal)										
	Q1		Q2		Q3		Q4		Q5		P_{trend}
	Adjusted mean SE										
Body mass index, kg/m^2											
Model 1	26.45	0.24	26.11	0.21	26.68	0.19	26.31	0.16	26.15	0.14	0.43
Model 2	27.03	0.37	26.72	0.35	27.11	0.33	26.59	0.32	26.14	0.33	0.0078
Waist circumference, cm											
Model 1	87.01	0.61	86.40	0.53	87.28	0.48	86.37	0.41	85.60	0.35	0.0451
Model 2	88.04	0.92	87.54	0.88	88.06	0.84	87.05	0.80	85.73	0.82	0.0042
C-Reactive protein, mg/L											
Model 1	0.95	1.06	0.97	1.06	1.01	1.05	0.90	1.04	0.78	1.04	0.0016
Model 2	0.94	1.10	0.98	1.10	1.03	1.09	0.91	1.09	0.81	1.09	0.05
Body fat, %											
Model 1	27.19	0.40	26.54	0.35	26.80	0.32	26.47	0.27	26.16	0.23	0.0355
Model 2	27.32	0.60	26.90	0.57	26.97	0.54	26.54	0.52	25.84	0.53	0.0043

Adjusted means and standard error of the mean (SE) presented. Orthogonal polynomial coefficients derived from median intake in each quintile were applied to estimate p-trend across quintiles of intake using general linear models. Model 1: age and sex. Model 2 adjusted for: age, sex ethnic category, marital status, final attained educational level, length or weekly working hours, smoking (current, previous, never), daily TV viewing hours and physical activity (IPAQ category) and continuous variable: alcohol intake (median g/day), continuous variables of mean daily intakes of: total energy, energy from saturated fat, polyunsaturated fat, non-milk extrinsic sugars (continuous variables). C-reactive protein log transformed for analyses.

Table S 8 Relationships between total fibre intake (g/1000kcal) and body mass index, waist circumference, C-reactive protein and body fat of Airwave Health Monitoring Study participants with low carbohydrate intake (<50% EI carbohydrate) (N = 4 541)

	Quintiles of energy adjusted total fibre intake (g/1000kcal)										
	Q1		Q2		Q3		Q4		Q5		P_{trend}
	Adjusted mean SE										
Body mass index, kg/m2											
Model 1	27.13	0.12	26.95	0.12	26.94	0.12	26.65	0.13	26.82	0.15	0.0467
Model 2	27.31	0.23	27.02	0.23	26.93	0.24	26.52	0.25	26.57	0.26	0.0001
Waist circumference, cm											
Model 1	89.21	0.29	88.37	0.30	88.22	0.31	87.43	0.33	87.61	0.39	0.0003
Model 2	89.47	0.57	88.57	0.58	88.31	0.59	87.39	0.62	87.40	0.65	<0.0001
C-Reactive protein, mg/L											
Model 1	1.11	1.03	1.02	1.03	0.98	1.03	0.89	1.03	0.86	1.04	<0.0001
Model 2	1.20	1.06	1.12	1.06	1.08	1.06	0.98	1.06	0.95	1.07	<0.0001
Body fat, %											
Model 1	28.40	0.19	27.93	0.19	27.81	0.20	27.22	0.21	27.53	0.24	0.0008
Model 2	28.46	0.35	28.09	0.36	27.91	0.36	27.22	0.38	27.49	0.40	0.0006

Adjusted means and standard error of the mean (SE) presented. Orthogonal polynomial coefficients derived from median intake in each quintile were applied to estimate p-trend across quintiles of intake using general linear models. Model 1: age and sex. Model 2 adjusted for: age, sex ethnic category, marital status, final attained educational level, length or weekly working hours, smoking (current, previous, never), daily TV viewing hours and physical activity (IPAQ category) and continuous variable: alcohol intake (median g/day), continuous variables of mean daily intakes of: total energy, energy from saturated fat, polyunsaturated fat, non-milk extrinsic sugars (continuous variables). C-reactive protein log transformed for analyses.

Table S9 and S10: Stratified by Goldberg classification of reporting – excludes participants reporting currently following a weight loss diet (n = 536)

Table S9 Under-reporters Estimated mean and body mass index, waist circumference, C-reactive protein and body fat by quintile of energy-adjusted total fibre intake (g/1000kcal) of Airwave Health Monitoring Study participants (n = 3,207)

	Quintiles of energy adjusted total fibre intake (g/1000kcal)										
	Q1		Q2		Q3		Q4		Q5		P_{trend}
	Adjusted mean SE										
Body mass index, kg/m2											
Model 1	27.73	0.16	27.51	0.16	27.55	0.15	27.18	0.14	26.96	0.15	<0.0001
Waist circumference, cm											
Model 1	92.7	0.72	92.01	0.71	91.91	0.69	91.52	0.69	90.86	0.73	0.0039
Model 2	90.9	0.40	90.60	0.40	90.34	0.39	90.52	0.39	90.12	0.41	0.0405
C-Reactive protein, mg/L											
Model 1	1.32	1.07	1.27	1.07	1.25	1.07	1.17	1.07	1.07	1.08	0.0008
Model 2	1.22	1.07	1.20	1.07	1.17	1.07	1.12	1.07	1.04	1.07	0.050
Body fat, %											
Model 1	30.1	0.42	29.63	0.42	29.58	0.41	29.36	0.41	29.13	0.43	0.009
Model 2	29.1	0.27	28.85	0.26	28.71	0.25	28.80	0.25	28.72	0.27	0.12

Adjusted means and standard error of the mean (SE) presented. Orthogonal polynomial coefficients derived from median intake in each quintile were applied to estimate p-trend across quintiles of intake using general linear models. Model 1 adjusted for: age, sex ethnic category, marital status, final attained educational level, length or weekly working hours, smoking (current, previous, never), daily TV viewing hours and physical activity (IPAQ category) and continuous variable: alcohol intake (median g/day), continuous variables of mean daily intakes of: total energy, energy from saturated fat, carbohydrate, polyunsaturated fat, non-milk extrinsic sugars (continuous variables). Model 2 additionally adjusted for body mass index (continuous). C-reactive protein log transformed for analyses.

Table S10 Acceptable energy intake reporters: Estimated mean and body mass index, waist circumference, C-reactive protein and body fat by quintile of energy-adjusted total fibre intake (g/1000kcal) of Airwave Health Monitoring Study participants (*n* = 3155)

	Quintiles of energy adjusted total fibre intake (g/1000kcal)										
	Q1		Q2		Q3		Q4		Q5		P_{trend}
	Adjusted mean SE										
Body mass index, kg/m^2											
Model 1	26.13	0.25	26.05	0.26	26.00	0.26	25.54	0.27	24.98	0.28	<0.0001
Waist circumference, cm											
Model 1	86.26	0.62	86.17	0.64	86.18	0.65	84.85	0.67	83.48	0.71	<0.0001
Model 2	85.68	0.35	85.69	0.36	85.78	0.36	85.34	0.38	85.04	0.40	0.0389
C-Reactive protein, mg/L											
Model 1	1.00	1.08	0.95	1.08	0.96	1.08	0.85	1.08	0.73	1.09	<0.0001
Model 2	0.98	1.07	0.93	1.07	0.94	1.07	0.87	1.08	0.78	1.08	0.0006
Body fat, %											
Model 1	26.41	0.44	26.38	0.45	26.14	0.46	25.24	0.47	24.71	0.50	<0.0001
Model 2	26.02	0.27	26.06	0.28	25.88	0.28	25.57	0.29	25.75	0.31	0.13

Adjusted means and standard error of the mean (SE) presented. Orthogonal polynomial coefficients derived from median intake in each quintile were applied to estimate p-trend across quintiles of intake using general linear models. Model 1 adjusted for: age, sex ethnic category, marital status, final attained educational level, length or weekly working hours, smoking (current, previous, never), daily TV viewing hours and physical activity (IPAQ category) and continuous variable: alcohol intake (median g/day), continuous variables of mean daily intakes of: total energy, energy from saturated fat, carbohydrate, polyunsaturated fat, non-milk extrinsic sugars (continuous variables). Model 2 additionally adjusted for body mass index (continuous). C-reactive protein log transformed for analyses, back transformed values presented.
